# How Does Problem Gambling Impact the Relationship Between Gambling Attitudes and Frequency?

**DOI:** 10.1007/s10899-025-10379-x

**Published:** 2025-03-06

**Authors:** Kim M. Caudwell, Angelica Fernandez Casanova, Mal Flack

**Affiliations:** 1 Faculty of Health, Charles Darwin University, Ellengowan Drive, Brinkin, NT 0810 Australia; 2 Researchers in Behavioural Addictions, Alcohol, and Drugs (BAAD), Charles Darwin University, Ellengowan Drive, Brinkin, NT 0810 Australia

**Keywords:** Gambling, Attitudes, Attitudes towards gambling scale, Problem gambling, Problem gambling severity index, Gambling frequency

## Abstract

Individuals’ beliefs and perceptions about gambling are known to influence gambling behaviours. However, the associations between gambling attitudes, gambling frequency, and problem gambling are unclear within the existing literature. The study aimed to elucidate the relationship between gambling attitudes, gambling behaviour, and problem gambling, using responses to the 2018 Northern Territory Gambling Prevalence and Wellbeing Survey. Data from 1629 participants (Mage = 49.87 years; SD = 14.58 years; 51.63% female) who completed the Attitudes Towards Gambling Survey (ATGS), Problem Gambling Severity Index (PGSI), and reported their gambling frequency were analysed. Prior to testing for moderation of the attitude-frequency path by PGSI risk category, the measurement model of the ATGS was assessed for invariance and one item was removed to improve its psychometric properties. Problem gambling severity moderated the relationship between attitudes towards gambling and gambling frequency. The relationship between attitudes towards gambling and gambling behaviour strengthened at higher levels of problem gambling. Results indicate that the higher the risk of problem gambling, the stronger the influence of gambling attitudes on gambling frequency. These findings are discussed in relation to cognitive dissonance, rationalisation, gambling motivation, and the potential implications for problem gambling prevention strategies.

Gambling is a popular activity among Australians, with 38% of Australian adults reporting gambling at least once a week (Australian Gambling Research Centre, 2023). While at the population level, most gamblers will not experience significant impacts from their gambling, others– including families and community members - may experience negative effects (Goodwin et al., 2017; Langham et al., 2016). As such, the term *problem gambling* is used to refer to gambling that leads to negative consequences and functional disruptions (Delfabbro, 2013). Importantly, problem gambling is not exclusive to those exhibiting clinical symptoms; it is estimated that twice as many people experience negative consequences from their gambling activities than meet clinical thresholds for gambling disorder (Australian Government Productivity Commission, 2010). As such, *gambling-related harm* is used to refer to the negative consequences of gambling activities which could be experienced more broadly– by the gambler themselves, but also by those connected to them (Delfabbro & King, 2019; Goodwin et al., 2017). Accordingly, gambling-related harm has attracted considerable attention from researchers attempting to quantify the impact of gambling at population levels, to shape policies and interventions that can reduce problem gambling and related harm (Browne et al., 2021; Goodwin et al., 2017).

## Attitudes Towards Gambling

Attitudes (i.e., evaluative beliefs) toward behaviours are influential determinants of behavioural engagement, and therefore are often primary targets in attempts to change people’s behaviour (Ajzen et al., 2018). Indeed, gambling advertising attempts to foster positive attitudes towards gambling, by normalising, glamorising, and incentivising gambling (Bouguettaya et al., 2020). On the other hand, public health and regulatory approaches may foster less positive or more negative beliefs about gambling, by conveying the risk of harm (Muñoz et al., 2013). Given attitude change confers small to medium effects in relation to health behaviour change (Sheeran et al., 2016), and prompts help-seeking behaviour (Rosen et al., 2020), interventions may bring about considerable reductions in problem gambling at the population level. Similarly, attitudes toward gambling are often used as a proxy for public support of gambling regulation and support programs (Donaldson et al., 2016).

Generally speaking, higher positive gambling attitudes correspond with higher gambling engagement (e.g., Canale et al., 2016; Flack & Morris, 2017; Kristensen et al., 2023; McAllister, 2014). Similarly, longitudinal research among adolescents has shown gambling attitudes become more positive as gambling participation increases (Pallesen et al., 2016). However, the relationship between attitudes and problem gambling appears more complex, with some studies reporting positive associations (e.g., Andrà et al., 2022; Canale et al., 2016; Zhou et al., 2019), negative associations (Donaldson et al., 2016; Dowling et al., 2021), or no association at all (Dixon et al., 2016). While there are various attitudinal measures of gambling throughout the literature, perhaps the most widely used measure is the Attitude Towards Gambling Scale (ATGS; Wardle, 2007). A recent systematic review by Kristensen et al. (2023) reviewed of the use of the ATGS across the available gambling literature, confirming that attitudes and gambling frequency are positively associated, and observing mixed findings in relation to associations between attitudes and problem gambling. The authors observe that those experiencing gambling problems are likely to be more frequent gamblers, and more frequent gamblers endorse more positive gambling attitudes. Although these findings are informative, the review was not able to determine the strength or the variability of the positive relationships between attitudes and gambling frequency or probe whether problem gambling severity influenced this relationship.

Elucidating the role of problem gambling in the attitude-frequency relationship may help to inform persuasion-based attitudinal change in public health and clinical settings, reducing gambling-related harm and the incidence of problem gambling. Notably, a recent meta-analysis of global gambling participation has estimated 8.7% of adults engage in any-risk gambling (i.e., engagement in any level of gambling risk behaviours), with a proportion of 1.4% engaging in problematic gambling (Tran et al., 2024). Given gambling-related messaging is intended to promote safer gambling at the population level, it is important to consider the effect of gambling attitudes on gambling frequency in relation to problem gambling (e.g., Newall et al., 2024). For instance, a recent meta-analysis by Joyal-Desmarais et al. (2022) outlines how the persuasiveness of messaging is dependent on the congruence of the message with a person’s underlying beliefs. Specifically, positive matching increases the effectiveness of persuasion (by an average effect size of *r* =.20), negatively matched messages may undermine persuasive effects in relation to attitude and subsequent behaviour change– or lead them to backfire. Indeed, “boomerang effects” have been observed in responsible gambling messaging for problem gamblers specifically, where messages appear to increase normative perceptions of gambling (De Jans et al., 2023). For instance, a responsible gambling message that suggests others are also experiencing gambling-related harm may lead a problem gambler to assume their own experiences are common.

The present study therefore aimed to ascertain the extent to which the gambling attitude-frequency relationship was dependent on the level of problem gambling, using a large-scale sample.

## Methods

### Procedure

A secondary data analysis was conducted using data from the Northern Territory (NT) 2018 Gambling Prevalence and Wellbeing Survey (Stevens et al., 2019). The original survey collected data from 5000 NT residents, aged 18 and over, via phone interview using a random selection of phone numbers. Prior to analysing the relationship between the variables and testing the moderation model, participants were excluded if they had not reported engaging in gambling activities over the last year, or did not complete the problem gambling screen. Analyses were conducted using IBM SPSS Statistics 29.0. Missing value analysis revealed < 5% of the attitude measure data were missing, with Little’s MCAR test non-significant: *χ*^2^(269) = 267.26, *p* =.519. Multiple imputation was therefore used for missing values, using an Expectation-Maximisation (EM) approach, leading to a sample of *N* = 1629.

### Measures

#### Problem Gambling

The Problem Gambling Severity Scale (PGSI) from the Canadian Problem Gambling Scale (Ferris & Wynne, 2001) was used to measure problem gambling. Nine items are scored on a four-point Likert scale (0 = never 1 = sometimes, 2 = most of the time, 3 = almost always), with four items referring to problem gambling behaviours (e.g., How often have do you bet more than you can afford to lose? ) and the other five referring to negative consequences of gambling (e.g., how often has your gambling caused you any health problems, including stress or anxiety? ), with the total score commonly used to assess problem gambling severity (Tseng et al., 2023). The PGSI scores also allow for classifying participants into different risk categories: 0 = no risk, 1–2 = low risk, 3–7 = moderate risk, ≥ 8 = problem gambling (Ferris & Wynne, 2001).

#### Attitudes Towards Gambling

Attitudes were measured with the 8-item version of the Attitudes Towards Gambling Scale (ATGS-8; Canale et al., 2016). The ATGS-8 is the shorter form of the original 14-item scale, which was designed to gain a broad idea of the attitudes towards gambling in general and to assess the opinions on gambling in the general community (Wardle, 2007). The ATGS-8 uses a five-point Likert scale where each item is endorsed from strongly agree (5) to strongly disagree (1). Four items are phrased to expressed negative attitudes towards gambling (e.g., gambling should be discouraged) and the other four phrased to express positive attitudes towards gambling (e.g., gambling livens up life); the latter items are recoded, meaning higher scores indicate more positive attitudes.

#### Gambling Frequency

Participants indicated the number of times they participated in lotteries, bingo, racetrack betting, casino table games, sports betting, raffles/sweeps, keno, pokies, instant scratchies, informal (e.g., card-based) and non-sports event betting (e.g., awards), and other gambling within the previous 12 months. The response provided for each activity was converted to number of days per year, and gambling frequency was calculated by summing the number of times a person engaged in all the activities over the previous year.

## Results

### Participants

Participants (*M*_age_ = 49.87 years, *SD*_age_ = 14.58 years; 51.63% female) were residents of the Northern Territory, with 16.82% identifying as Aboriginal or Torres Strait Islanders, and the majority of the participants (94.17%) reporting speaking English as their household main language. Statistics for 12-month frequency by gambling type, across age, sex, and PGSI risk status are included in Table 1.

### Preliminary Analyses

The factor structure of the ATGS-8 was initially examined to ensure consistency between those exhibiting no risk of problem gambling (i.e., PGSI = 0), to those with any risk of problem gambling (i.e., PGSI ≥ 1), consistent with previous research (Donaldson et al., 2016). An invariance testing approach was undertaken using confirmatory factor analyses in IBM SPSS AMOS v. 28, involving testing successive restrictions on the measurement model (i.e., configural, metric, and scalar invariance), and assessing for reductions in model fit. The adopted threshold for invariance was where the associated decrement in CFI was less than 0.010, and in RMSEA less than 0.015 (Chen, 2007). When assessing scalar invariance, high standardised residual covariances implicated the ATGS-8 item 7 (i.e., “gambling livens up life”), with a *t*-test confirming that mean scores on this item were statistically significant for the risk categories (*p* <.001). The invariance test was conducted with the item removed, and the modified scale (i.e., “ATGS-7”) was tested for model fit and invariance, deemed acceptable^1^ and used in subsequent analyses. Model results for both ATG-8 and ATG-7 are included in Table 2.

**Table 1:** Descriptive statistics for gambling frequency by type, across age, sex, and PGSI risk (i.e., PGSI < 1, PGSI ≥ 1)

Variable	Category	*n*	Pokies		Racing		Scratchies		Keno		Lotto		Bingo		Casino games		Sports betting	
			*M*	*SD*	*M*	*SD*	*M*	*SD*	*M*	*SD*	*M*	*SD*	*M*	*SD*	*M*	*SD*	*M*	*SD*
Age range	18–34	274	15.54	46.42	30.63	84.85	7.35	11.67	17.24	46.81	31.53	212.81	9.11	16.53	5.34	7.41	23.82	40.14
	35–49	499	17.57	31.98	18.90	43.58	10.20	34.90	22.67	52.48	23.34	34.69	13.30	34.33	3.82	6.58	22.16	36.80
	50–64	596	24.26	58.75	31.89	67.38	12.64	18.62	25.92	47.65	35.50	128.35	27.58	28.83	9.25	18.77	28.23	40.13
	65+	260	25.97	35.58	34.19	83.20	28.72	38.54	30.55	50.76	36.99	42.30	43.44	54.52	2.88	3.72	11.20	11.22
Sex	Male	788	23.77	53.36	42.09	80.31	16.09	37.42	30.17	56.95	35.54	117.50	9.00	19.05	6.85	12.36	25.45	37.36
	Female	841	18.58	39.75	7.62	28.96	11.34	19.93	18.06	39.46	27.77	114.04	23.40	37.04	2.33	2.64	14.74	38.99
PGSI Risk	No risk	1115	15.97	37.89	19.33	50.75	13.11	23.80	20.38	40.65	31.36	138.02	20.33	33.24	4.52	11.58	23.01	36.04
	At risk	514	25.95	53.30	39.35	81.92	13.45	33.48	30.41	60.39	32.18	45.02	24.29	40.38	6.34	9.71	24.05	39.34

Note. Frequencies for informal betting, non-sports event betting, and ‘other’ gambling are excluded due to low frequencies across categories

**Table 2:** Measurement model invariance tests comparing the PGSI ‘no risk’ category subsample to the PGSI ‘any risk’ subsample for the ATGS-8 and ATGS-7

	Sample / Invariance	χ^2^	df	CFI	RMSEA	∆CFI	∆RMSEA
ATGS-8	Full sample (*n* = 1629)	197.68	20	0.939	0.065	-	-
	No risk (*n* = 1115)	154.5	20	0.937	0.078	-	-
	Any risk (*n* = 514)	69.51	20	0.937	0.069	-	-
	Configural invariance	224.01	40	0.937	0.053	-	-
	Metric	228.99	47	0.938	0.049	0.001	0.004
	Scalar	293.85	55	0.919	0.052	0.019	0.003
ATGS-7	Full sample	107.13	14	0.961	0.064	-	-
	No risk	78.57	14	0.964	0.064	-	-
	Any risk	44.58	14	0.951	0.065	-	-
	Configural	123.51	28	0.961	0.046	-	-
	Metric	127.27	34	0.961	0.041	< 0.001	
	Scalar	140.04	41	0.959	0.039	0.003	

Note. ATGS-7 = Attitudes Towards Gambling Survey 7-item; PGSI = Problem Gambling Severity Index. Criteria for model fit: CFI > 0.95, RMSEA < 0.08. Criteria for invariance testing, ∆CFI < − 0.01; ∆RMSEA < − 0.015 (Chen, 2007)

Descriptive statistics and correlations are shown in Table 3. Overall, the respondents reported unfavourable attitudes towards gambling (*M* = 21.01, *SD* = 5.12). A correlational analysis indicated a positive relationship between gambling attitude and gambling frequency (*r* =.16, *p* <.001); no significant correlation between PGSI score an ATG7 was observed (*p* =.899).

**Table 3:** Descriptive statistics and correlations between variables for the sample

Variable	Min	Max	M	SD	1	2	3	4
1. ATG7	7	35	18.54	4.54	0.77			
2. PGSI	0	22	0.88	2.14	− 0.02	0.81		
3. Frequency	1	2706	59.10	129.31	0.16**	0.26**	-	
4. Age	18	92	49.87	14.58	0.02	− 0.10**	0.08*	-
5. Gender			-	-	− 0.19**	− 0.07*	− 0.12**	− 0.03

Note. ATGS-7 = Attitudes Towards Gambling Survey 7-item; PGSI = Problem Gambling Severity Index. Gender was coded 1 (male) and 2 (female). *p <.01. **p <.001. Cronbach’s α values are included along the principal diagonal

### Moderation Analyses

**Fig. 1:**
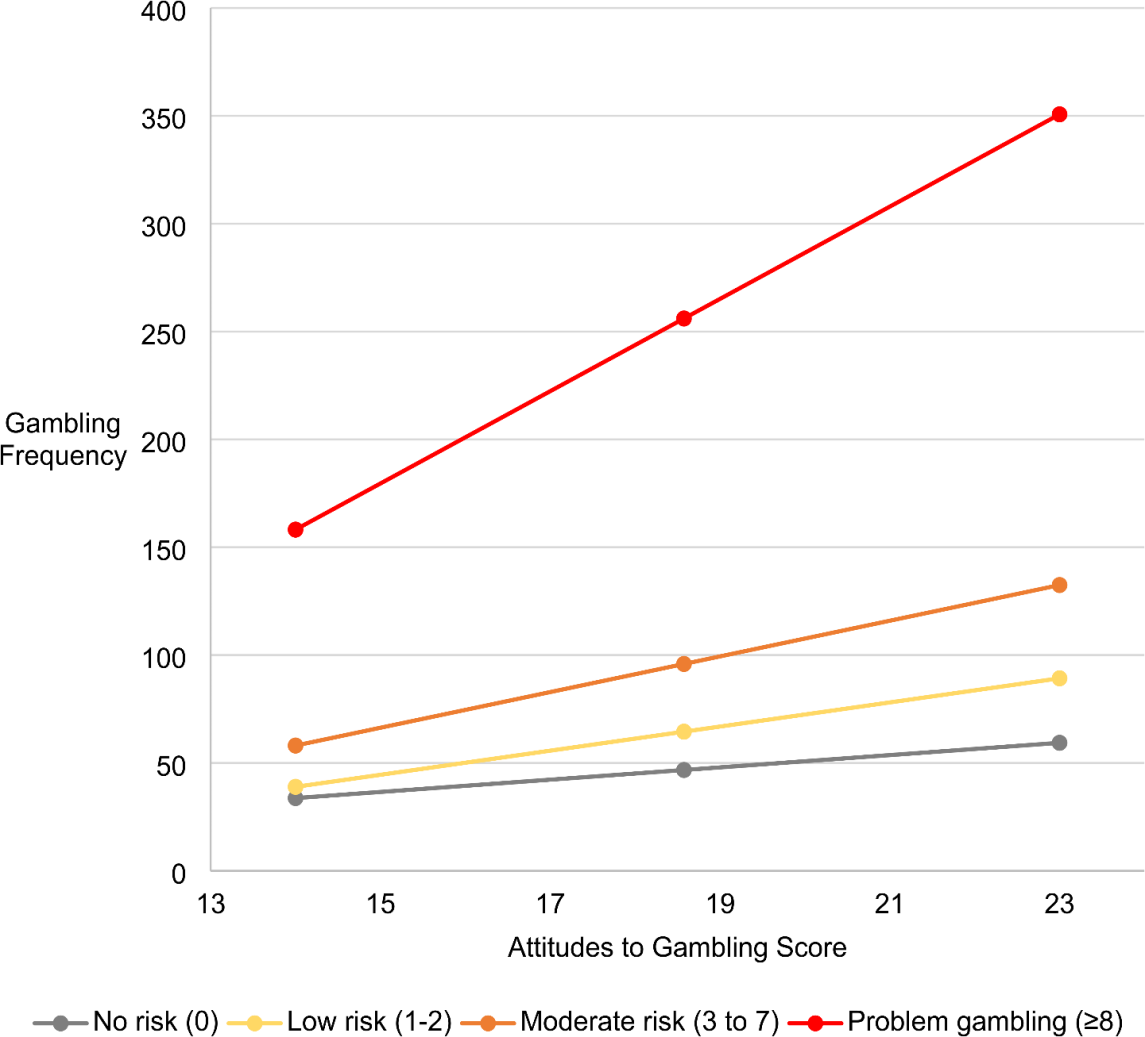
Plot of regression slopes at different PGSI risk categories

To test the hypothesis that problem gambling would moderate the relationship between attitudes towards gambling and gambling frequency, the PROCESS v4.3 Macro (Hayes, 2018) was used (i.e., Model 1), with the PGSI total score as the moderating variable, and age and gender as covariates. As the PGSI can be used to differentiate levels of risk, Helmert coding was used to probe the interaction at the no risk (*n* = 1115), low risk (*n* = 342), moderate risk (*n* = 131) and at risk (*n* = 41) categories, to facilitate comparison of the means for each group with those ordinally higher (Hayes & Montoya, 2017). Overall, the model accounted for 10.87% of the variance in gambling frequency, *F* (9, 1604) = 21.74, *p* <.001. Results of the Helmert approach to probing the moderation effect are included in Table 4, with a visual depiction of the interaction included in Fig. 1.

**Table 4:** Results of helmert contrasts for the moderating effect of PGSI risk category on gambling Attitude-Frequency path

Contrast (PGSI Score)	b	SE	t	*p*	95% CI
0 vs. ≥ 1	8.90	1.98	4.50	< 0.001	[5.01, 12.79]
*1–2 vs. ≥ 3*	9.24	3.01	3.07	0.002	[3.34, 15.14]
*3 to 7 vs. ≥ 8*	13.12	5.21	2.52	0.012	[2.90, 23.35]

Note. *R*^2^ = 0.11, *F* (9,1604) = 21.58, *p* =.002

## Discussion

The current study attempted to further elucidate the association between attitudes to gambling and gambling frequency, taking into account the experience of problem gambling. Specifically, the study aimed to test the hypothesis that problem gambling would moderate the effect of attitudes on gambling frequency. Results indicated two key findings: (1) the positive association between attitudes and frequency were amplified in higher problem gambling risk categories, while the relationship between attitudes and problem gambling was not significant; (2) that when comparing no risk to any risk problem gamblers, the ATGS-8 appears non-invariant. These findings require further discussion and illustrate the importance of considering problem gambling in relation to the measurement of attitudes and work to establish associations between attitudes and gambling frequency (Kristensen et al., 2023).

The finding that attitudes towards gambling are associated with gambling frequency is consistent with previous research (Canale et al., 2016; Flack & Morris, 2017; McAllister, 2014). While these findings support the notion that changing gambling attitudes may change gambling frequency, the presence of problem gambling as a moderator indicates that attitude-behaviour relationships should not be investigated without considering problem gambling severity.

There are a range of plausible interpretations for this moderation effect. One example is *cognitive dissonance* (Festinger & Carlsmith, 1959)– the distress experienced by an individual when their beliefs and behaviours are incongruent. Cognitive dissonance is thought to create a motivational impetus to change one’s attitudes or behaviour to alleviate distress (Cooper, 2019), which may facilitate avoiding disconfirming information, and engaging in motivated reasoning (e.g., Gillespie, 2020). Potentially, as people with positive gambling attitudes experience gambling-related harm, instead of gambling less frequently, they might rationalise their gambling experiences to maintain overall favourable attitudes towards gambling. Indeed, rationalisation has been noted as a form of dissonance management in qualitative research with EGM players (Hahmann & Monson, 2021). Similarly, the tendency to rationalise continued gambling despite accruing mounting losses may also be partly explained by the motives or expectancies underlying gambling, which are known to differ between non-problem and problem gamblers. For instance, gambling to escape or manage undesired emotional states is consistently associated with problem gambling (Alaba-Ekpo et al., 2024; Lee et al., 2024).

Though speculative, the experience of cognitive dissonance and rationalising continued gambling due to the anticipated emotional benefits may maintain positive attitudes at higher levels of problem gambling risk and is worth exploring further in public health and clinical contexts. When the immediate perceived benefits of gambling outweigh the perceived losses, positive attitudes may remain entrenched and resistant to conventional harm reduction messaging (De Jans et al., 2023; Delfabbro & King, 2020). Strategies aimed at changing attitudes may do well to induce dissonance (e.g., Wan & Chiou, 2010), and educate gamblers on the tendency to resolve cognitive dissonance through rationalisation, which may be delivered as part of a completement of techniques that constitute just-in-time adaptive interventions that incorporate dynamic tailoring (Dowling et al., 2023).

A different, but equally important finding, was that the ATGS-8 was not invariant between PGSI no risk and risk categories. This was unanticipated and has to the authors knowledge not been formally explored, and warrants some consideration. That the removed item (“gambling livens up life”) improved the fit of the ATGS implies it is somewhat conceptually distinct from other items (i.e., that are largely framed around the extent to which gambling should be controlled, or whether or not it is good or bad overall). It may be that individuals at any level of risk for problem gambling may interpret an item framed around improving life akin to a motive or outcome expectancy; such as anticipation that gambling will alleviate negative affect (Caudwell et al., 2024), or otherwise manage disordered mood symptoms (Vaughan & Flack, 2022). While the ATGS-8 has been found to be gender invariant, this study’s findings indicate its structure should be investigated in relevant contexts, especially with respect to gambling modality and motives (Richardson et al., 2023). More targeted research in this space would allow for the refinement of the ATGS and improve its relevance and interpretation in a variety of research contexts.

Given the inconsistencies in gambling attitude-problem gambling correlations throughout the extant literature (Kristensen et al., 2023), the present findings again highlight the importance of considering contextual elements that may influence the formation of attitudes, engagement with gambling behaviours, and experience of problem gambling severity, and conclusions about their interrelationships. For instance, research in younger samples (e.g., Dixon et al., 2016) may introduce floor effects where individuals are restricted from engaging in higher risk forms of gambling); similarly, university student samples appear to hold more negative attitudes towards gambling and be at lower risk of gambling problems than those in the general population (Gainsbury et al., 2014). Of note too, is that publicly-sampled attitudes toward gambling may be generally negative when participants conflate gambling with problem gambling (Delfabbro & King, 2021). This is especially important, given the role of the ATGS in capturing attitudes that may change as a result of policy or public scrutiny on gambling regulation (e.g., Donaldson et al., 2016). Future research may also need to investigate attitude-frequency associations in purposive, higher risk contexts (e.g., treatment-seeking samples) or subsamples from population-based research (e.g., age groups, gender), or with focus on preferred gambling type, and pursue longitudinal designs that may complement the pathways approach to problem gambling (Billieux et al., 2022; Nower et al., 2022). Such research can inform attitude and behaviour change interventions that may be dependent on problem gambling status.

### Limitations and Future Research

While this study makes an important contribution to the attitude-gambling frequency literature, some limitations warrant consideration. Firstly, the scale of prevalence studies necessitates the use of self-report measures, yet introduces issues in relation to participant accuracy of reported gambling behaviours and problem gambling retrospectively (i.e., recalling behaviour over the last 12 months). Secondly, the study’s cross-sectional design precludes definitive statements about causality. Future research should adopt longitudinal designs to consolidate understanding of the patterns of gambling attitude development, and influence on gambling behaviour, over time. Finally, the study’s findings are based on a population-based survey of the Northern Territory of Australia, which are undoubtedly shaped by specific cultural and legislative dynamics, which limits the generalisability of the findings to other contexts. Future research, especially that involving large-scale prevalence studies, should attempt to replicate the findings– particularly those in relation the attitude measurement invariance– in other national and cultural contexts.

## Conclusion

The present study shows that the investigation of the association between gambling attitudes and frequency needs to consider problem gambling risk. Specifically, the attitudes of those who are in higher risk problem gambling categories appear to have a more pronounced effect on gambling frequency. The findings of this study carry important implications for the measurement of gambling attitudes in different contexts, especially in relation to the measurement of attitudes in problem gamblers. They also indicate that attitude change among problem gamblers may need to afford a greater role to cognitive dissonance and motivation for gambling, and the propensity of problem gamblers to rationalise their experiences and maintain overall positive attitudes toward gambling.

## Footnotes

1 Scalar invariance was essentially confirmed; though the CFI decreased slightly, the RMSEA, which is arguably more sensitive to noninvariant models in larger samples (i.e., *N* > 300; Chen, 2007), remained well within the recommended threshold.
